# Using ancestral information to detect and localize quantitative trait loci in genome-wide association studies

**DOI:** 10.1186/1471-2105-14-200

**Published:** 2013-06-20

**Authors:** Katherine L Thompson, Laura S Kubatko

**Affiliations:** 1 Department of Statistics, The Ohio State University, Columbus, OH 43210, USA

**Keywords:** Phylogenetic analysis, Genome-wide association study (GWAS) data, Stochastic processes, Coalescent theory, Ornstein-Uhlenbeck process

## Abstract

**Background:**

In mammalian genetics, many quantitative traits, such as blood pressure, are thought to be influenced by specific genes, but are also affected by environmental factors, making the associated genes difficult to identify and locate from genetic data alone. In particular, the application of classical statistical methods to single nucleotide polymorphism (SNP) data collected in genome-wide association studies has been especially challenging. We propose a coalescent approach to search for SNPs associated with quantitative traits in genome-wide association study (GWAS) data by taking into account the evolutionary history among SNPs.

**Results:**

We evaluate the performance of the new method using simulated data, and find that it performs at least as well as existing methods with an increase in performance in the case of population structure. Application of the methodology to a real data set consisting of high-density lipoprotein cholesterol measurements in mice shows the method performs well for empirical data, as well.

**Conclusions:**

By combining methods from stochastic processes and phylogenetics, this work provides an innovative avenue for the development of new statistical methodology in the analysis of GWAS data.

## Background

The goal of quantitative trait mapping based on genome-wide association study (GWAS) data is to find single nucleotide polymorphisms (SNPs) that are associated with a set of quantitative trait (or phenotypic) values under study. Many quantitative traits are thought to have both a genetic basis and an environmental basis, making the associated genes difficult to identify from genetic data alone. The biological complexity of the evolutionary history of genes and the environmental factors acting simultaneously on the trait values makes this a challenging task, even with very large data sets.

Quantitative trait mapping has two distinct goals, detection and localization. Detection is achieved if any SNP in a certain region is found to be significant during the association study, while localization addresses how close the detected SNP(s) are to the true causative SNP(s). Localization is usually measured by distance between a significant SNP and the true SNP. Since most data sets will include a large number of SNPs, it is very unlikely that any statistical method will pick up a truly causal SNP, but clearly methods that can provide relatively precise localization will be the most useful.

Methods of quantitative trait mapping can be broadly classified into two groups: those that model the shared evolutionary history, usually in the form of a phylogenetic tree, and those that do not. Non-tree based methods used in quantitative trait mapping include methods that analyze each marker independently (e.g. the *t*-test), and those that analyze groups of markers together (e.g., Haplotype Association Mapping [1] and Single Marker Analysis [2]). The *t*-test simply groups samples according to allele type at each SNP, and uses a two-sided alternative to look for a significant difference in mean trait value between groups. Both Haplotype Association Mapping (HAM) and Single Marker Association (SMA) perform an ANOVA on particular groupings of samples to assess the significance of the groupings [2]. Since these methods fail to consider the evolutionary relationships among SNPs, they may have difficulty detecting some associations between SNPs and quantitative traits. This leaves room for improvement in the power of detection of associated SNPs. In fact, it has recently been noted that the application of a phylogenetic framework to analysis of GWAS data may be beneficial [3].

By using information contained in the relationships among SNPs, tree-based methods gain power in detection and localization. However, this gain in power comes at the expense of an increased computational cost. In spite of the computational issues, many tree-based methods have recently been proposed for this problem. In the case of discrete trait data, these methods include LATAG, implemented in the software TreeLD [4], MARGARITA [5], and Blossoc [6]. For quantitative traits, tree-based methods in common use include TreeQA [7], QBlossoc [8], and HTreeQA [2]. Several of these methods (Blossoc [6], TreeQA [7], and QBlossoc [8]) are based on the idea of *local perfect phylogenies*, which are phylogenies built on sets of neighboring compatible SNPs identified by the four-gamete test. These methods also require that the SNP data be phased into haplotypes prior to analysis, which is a nontrivial task. The HTreeQA method [2] avoids this difficulty during analysis by using a tri-state semi-perfect phylogenetic tree, which can be built on unphased genetic data.

Tree-based techniques must assume an underlying model to represent the genealogical history among SNPs along a chromosome. The most common model for evolutionary relatedness within a population is the coalescent process [9,10]. At a single locus, the coalescent process describes the genealogical history among sampled individuals in the form of a phylogenetic tree. In the case of GWAS data, however, the two competing processes of coalescence and recombination are occuring simultaneously along a chromosome, and a single phylogenetic tree cannot be used to model the genealogical history among all individuals for the entire chromosome. When recombination occurs between two genetic sequences, the sequences exchange genetic material at a recombination point, leaving a situation where the portion of the genetic sequence on one side of the recombination point is the same as the sequence present before the recombination, while the portion of the sequence on the remaining side is new [11]. Although phylogenetic trees model genetic sequence data well in the absence of recombination events, perfect phylogenetic trees do not exist to model incompatibilities in genetic data on both sides of a recombination point simultaneously. In this case, the genealogical history of a chromosome can be represented by an Ancestral Recombination Graph (ARG), a phylogenetic network representing both recombination and coalescent events [12]. ARGs provide a model that accommodates the fact that while a (true) local tree exists at each site along the chromosome, neighboring trees may be incompatible due to recombination events. ARGs represent these clusters of incompatible local trees, and can be used to determine the marginal tree at each SNP along the chromosome [12]. However, ARGs can be difficult to estimate from SNP data [see, for e.g., [4] and references therein]. Methods have thus been proposed to estimate the important features of ARGs for particular applications. Many of the methods used in the GWAS setting replace estimation of the entire ARG by estimation of the marginal phylogenies at each SNP. This will be the approach we use here.

The tree-based methods mentioned above vary in the way that the phylogenetic information is used in the subsequent analysis. One method of particular interest to the present study is QBlossoc [8]. After local phylogenies are estimated for each SNP, QBlossoc uses this information to partition the sampled individuals into some number of clusters, *k*, and calculates a score for each possible set of *k* clusters defined by the phylogenetic tree. The score calculated is a penalized likelihood (the penalty is determined by the number of clusters), where the likelihood is a multivariate normal with a different mean in each cluster and an overall shared variance, with zero covariance among observations. The maximum score over all possible sets of *k* clusters defined by the phylogeny is used to assess the significance of each SNP. This technique produces a test statistic at each location along the genome. Although this clustering technique accounts for the shared evolutionary history among SNPs, QBlossoc has two weaknesses rooted in its assumptions during the score calculation; namely, QBlossoc assumes both independence and a common variance among the quantitative trait values. The method proposed here is a modification of QBlossoc that addresses these two weaknesses.

**Figure 1:**
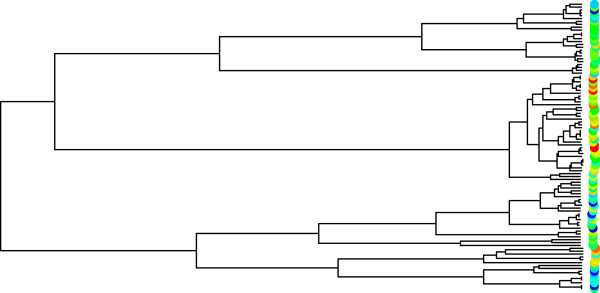
**Phylogenetic tree at an associated SNP.** This tree shows the evolutionary history of a SNP for 50 diploid observations of a quantitative trait (colored circles on right). The low values of the trait are blue, and the high values are red. The clustering pattern shown by the tree is an indication of the association between the SNP and the trait.

Our proposed data analysis technique uses the same near local perfect phylogenies built by [6], but also estimates the branch lengths of each marginal tree via a modification of the algorithm from [13]. Estimating the branch lengths enables estimation of the variance-covariance structure of the data using a Brownian Motion model for trait values along the tree [14]. This modeling choice allows the covariance between two observations to be proportional to the length of their shared evolutionary history. Our score statistic is also a penalized likelihood, where the likelihood is a multivariate normal likelihood for the observations using the same mean structure as QBlossoc, but with variance-covariance structure determined by the estimated phylogeny. We find that the estimation and use of the variance-covariance structure is especially important in the presence of strong population structure among the observations. For instance, in the example data in Figure 1, the SNP clearly has an effect on the trait value, but the evolutionary history is also very important. Due to the mixture that occurs both early and late in the evolutionary history of the SNP, assuming the resulting observations are independent among the subpopulations could hinder the ability to detect the association between this SNP and the quantitative trait.

Here, we propose a data analysis method that accounts for the covariance structure present in GWAS data sets, and show that it generally performs similarly to QBlossoc in terms of power of detection and localization, with strong performance in the presence of population structure. Finally, the proposed data analysis method is applied to a GWAS data set containing SNP data for 288 outbred mice [15]. Phenotypic data for each mouse includes observations of eight quantitative cardiovascular traits. The SNP sites on two chromosomes with previously-detected strong signals and one chromosome without a previously-detected strong signal are analyzed.

## Methods

Since the goal is to search for SNPs associated with a quantitative trait, we will consider both detection and localization. The proposed analysis technique includes calculation of a score at each SNP site and an assessment of significance by performing hypothesis tests via permutation. In order to examine the performance of the methods, we use a novel data simulation technique so that we know the location of the SNP truly associated with the quantitative trait (if one exists). This yields an opportunity to compare the type I error and power of the proposed method with that of QBlossoc. We begin by giving the details of the method of analyzing the data, and then describe the simulation technique.

### Data analysis

The evolutionary history at each SNP site can be represented by a local phylogenetic tree, *Θ*. At each SNP, the local tree topology is estimated using Blossoc’s approach [6]. The branch lengths of each tree are estimated using a modification of the algorithm in [13], which yields an approximation to the maximum likelihood estimate of the branch length. In the case of DNA sequence data, the Rogers-Swofford method [13] is based on the use of a fast heuristic method to approximate the state at each internal node of the tree. The distance between a pair of nodes in the tree, $\hat{p}$, is then calculated to be the proportion of the sites in the sequence that differ between the reconstructed states at each node. $\hat{p}$ can then be used to obtain an estimate of the branch length under an appropriate model, such as the Jukes-Cantor model [16].

We modify this method to handle SNP data as follows. First, the same heuristic method as in the Rogers-Swofford method (based on a most parsimonious reconstruction) is applied to the phased 0-1 SNP data at each internal node of the tree. The proportion of SNPs that differ between each node, $\hat{p}$, is counted, and the branch length connecting the two nodes is estimated to be:

(1) $$\begin{array}{lcrr} \hat{d}=-\frac{1}{2}\ln(1-2\hat{p}) & & & \end{array}$$

If $\hat{p}=0$ for a branch, then we set $\hat{p}=\frac{1}{\text{number of SNPs}}$. This distance equation is derived under the M2 model, a two-state Markov model for nucleotide sequence data, which is a specific case of the more general Mk model described in [17]. Notice that the branch length estimate, $\hat{d}$, increases as the proportion of differing SNPs between two nodes increases, as expected.

**Figure 2:**
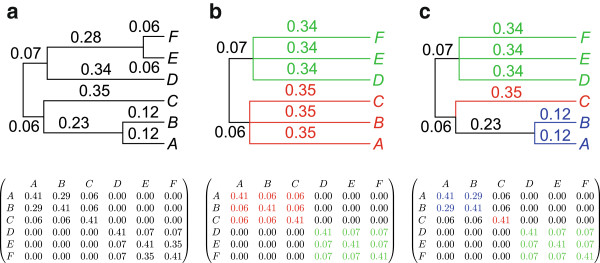
**Example of a six-taxon tree with branch lengths.** The overall tree, *Θ*, along with its variance-covariance matrix, *V*(*Θ*), is shown in (**a**). The corresponding clustered tree along with the variance-covariance matrices, *V*(*Θ*), for *k*=2 and *k*=3 clusters are in (**b**) and (**c**), respectively.

For each estimated local phylogeny, the branch lengths are used to estimate the variance-covariance matrix of the tree, *V*(*Θ*), as shown in the example in Figure 2. The covariance between two quantitative trait values is defined to be proportional to the time of shared evolutionary history between those two observations. Given the local estimated phylogeny at the SNP of interest, the quantitative trait data were assumed to follow a multivariate normal distribution, with covariance structure determined by the local phylogeny.

The proposed method accounts for only a focused portion of the evolutionary history among observations using a clustering technique. At each SNP site, the tree can be partitioned into *k* clusters using only the earliest (*k*−1) edges in the tree. An example of this clustering is shown in Figure 2. For a fixed partition of the tree into *k* clusters, we define a matrix, *D*, with elements:

(2) $$\begin{array}{llll} D_{\text{ij}}=\left\{\begin{array}{l} 1,\;\text{if observation}i\;\text{falls in cluster}\;j\\ 0,\;\text{otherwise} \end{array}\right) & & & \end{array}$$

for *i*=1,2,…,*n* and *j*=1,2,…,*k*, where *n* is the number of observations (for diploid individuals, this is twice the number of individuals in the study). Then the trait data, ***Y***=(*Y*_1_,*Y*_2_,…,*Y*_*n*_), are assumed to follow a multivariate normal distribution along tree *Θ*:

(3) $$\begin{array}{lcrr} Y∼N(D\mu ,\sigma^{2}V) & & & \end{array}$$

Here, as in QBlossoc, each cluster has its own mean, denoted ***μ***=(*μ*_1_,*μ*_2_,…,*μ*_*k*_). However, instead of assuming independence, the variance-covariance matrix of the tree, *σ*^2^*V*=*σ*^2^*V*(*Θ*), allows for covariance structure to be present among the quantitative trait observations.

Using the distribution in Equation 3, the maximum likelihood estimates of the parameters are straightforward to calculate,

(4) $$\hat{\mu }={\left(D^{T}V^{-1}D\right)}^{-1}D^{T}V^{-1}Y$$

(5) $${\hat{\sigma }}^{2}=\frac{{\left(Y-D\hat{\mu }\right)}^{T}V^{-1}(Y-D\hat{\mu })}{n}$$

Hypothesis testing is carried out using a likelihood framework. In particular, we use a penalized likelihood similar to that proposed by [8]. We define the Likelihood Score Statistic (LSS) to be

(6) $$\begin{array}{lcrr} \text{LSS}={\text{max}}_{k}\{2\;\text{lnL}(\hat{\mu },{\hat{\sigma }}^{2}|Y,\Theta ,V)-k\ln(n)\}. & & & \end{array}$$

To calculate LSS, the maximum likelihood is penalized by subtracting a penalty as in the Bayesian Information Criterion (BIC). Calculation of the likelihood involves estimation of (*k*+1) parameters, including the mean trait value in each cluster and the variance, *σ*^2^. The BIC criterion penalizes for *k* of these parameters. At each SNP, a local tree is scored according to (6), for varying numbers of clusters, *k*=1,…,*k*_*max*_, and the resulting tree score is the maximum score over the number of clusters.

To address detection, after the score in Equation 6 is calculated for the phylogenetic tree at each locus along a chromosome, permutation testing based on this location-specific test statistic can be used to evaluate significance. Permutation of the observed trait values among the tips of the estimated phylogenetic tree yields permuted data sets, and the score in Equation 6 is calculated for each permuted data set. The p-value for detection at each locus is the proportion of data sets scoring higher than the observed data set at each particular locus. To address localization, the distance (in DNA base pairs) between the maximally-scored locus and the disease locus is calculated.

In addition to the tree topology, our method also requires an estimate of the covariance structure in the data. This covariance structure is estimated via estimation of the branch lengths along the topology. By using the clustered tree to consider only the broad-scale phylogenetic relationships among SNPs, our technique can account for the evolutionary history among genes without using all coalescent relationships. Using only broad-scale relationships enables a computationally feasible algorithm that is able to account for the most important aspects of the covariance structure among observations.

### Data simulation

To assess the performance of the proposed likelihood technique, simulated data sets are used. This provides a setting where the presence and location of the SNP truly associated with the quantitative trait is known. In our simulation study, we simulate SNP data for 100 replicate data sets from a diploid population using the program ms (without selection) [18]. Each data set consists of the SNP data corresponding to one chromosome. For each simulated replicate, a single DNA base pair location is randomly chosen to be associated with the trait. This choice of “disease” locus is restricted so that the minor allele frequency is between 10% and 30%.

For each SNP, quantitative data is simulated along the phylogenetic tree at the disease locus according to a generalized version of the Ornstein-Uhlenbeck (OU) process described by [19],

(7) $$dY_{i}(t)=\alpha \left(\theta -Y_{i}(t)\right)\text{dt}+\sigma_{Y}dB_{i}(t)$$

where *Y*_*i*_(*t*) is the quantitative trait value for the *i*^*th*^ lineage at time *t*, *Θ* is the target trait value, *α* is the strength of selection toward the target value, *σ*_*Y*_ is the standard deviation of the process per unit time, and *dB*_*i*_(*t*) represents a Brownian Motion process for lineage *i*, so that values of *dB*_*i*_(*t*) for small time increments, *dt*, are independent, identically distributed random variables from a normal distribution with mean zero and variance *dt*. Thus, the OU process is a mean-reverting process with a deterministic component, *α*(*θ*−*Y*_*i*_(*t*))*dt*, modeling the selection of a trait toward the optimum target value, and a stochastic component, *σ*_*Y*_*dB*_*i*_(*t*), providing the “random noise” for the process. Notice that the deterministic portion of this process implies that the selection of the trait toward the target is proportional to the distance between the trait and the target value, *Θ*. When two observations share a portion of their evolutionary history, they share the trait value, *Y*_*i*_(*t*), for that portion of time. Along the corresponding phylogenetic tree, observations share an evolutionary history when they evolve along the same lineage.

When this process is applied in the setting of phylogenetics, the stochastic process gives the same value during the time when the evolutionary history is shared for any two observations. However, after two lineages split, their trait values evolve independently from one another. This implies that before a split, two observations are perfectly correlated, while after the split, they evolve in an uncorrelated manner.

For this study, a more flexible form of the Ornstein-Uhlenbeck process is used, the Generalized Hansen model [19]. This allows the trait to evolve toward a non-constant optimum (target) as follows,

(8) $$\begin{array}{lcrr} dY_{i}(t)=\alpha \left(\theta_{i}(t)-Y_{i}(t)\right)\text{dt}+\sigma_{Y}dB_{i}(t) & & & \\ {\;\theta }_{i}(t)=\left\{\begin{array}{c} \theta_{1},\;\text{if}\;\;X_{i}(t)=0\\ \theta_{2},\;\text{if}\;\;X_{i}(t)=1 \end{array}\right) & & & \end{array}$$

The trait is simulated according to this stochastic process with the target trait value determined solely by the SNP state at any time in the evolutionary history at that SNP, where *X*_*i*_(*t*) is the SNP state for the *i*^*th*^ observation at time *t*.

Using the Generalized Hansen Model leaves us with a quantitative trait value for each haplotype that has both a (deterministic) genetic component, determined by the SNP, and a stochastic component. This process imposes an evolutionary history of the quantitative trait which can be portrayed by the phylogenetic tree at the disease locus, and allows the two haplotypes of a diploid individual to evolve independently along the phylogeny at the disease locus. This is intuitive as long as two haplotypes for an individual are unrelated to the trait. In order to simulate data for each individual, or diplotype, based on the haplotypic data, we use an additive model. The trait value for each diplotype is the average trait value across the two copies of the trait for each individual at the disease location.

During the simulation studies, we simulate the SNP using these parameters in ms: the diploid population size was *N*_0_=20,000, the neutral mutation rate for each DNA base pair was *μ*=2.0×10^−10^, the rate of recombination per generation per DNA base pair was *ν*=10^−8^, and each simulated chromosome was 1,000,000 base pairs long. During simulation of the quantitative trait values, we vary the strength of selection, *α*, and the standard deviation of the quantitative trait per unit time, *σ*_*Y*_. The two target trait values we consider are *θ*_1_=80 and *θ*_2_=100.

## Results

### Simulation studies

In order to assess the performance of the proposed technique in terms of power and type I error, we begin by analyzing data sets which either include a truly-associated SNP, or do not include an associated SNP. These data sets are simulated using the technique described above. After simulating the data sets for specified parameter values, the local phylogenetic tree at each SNP is estimated using Blossoc, and branch lengths are estimated as described previously. Next, the score in Equation 6 is calculated, using *k*_*max*_=15. The same technique is applied to 200 permutation data sets created by using a permutation of trait values across individuals. The type I error, power, and localization for QBlossoc and LSS are presented in Tables 1 and 2.

**Table 1:** Type I error for simulated data sets

**Parameters**		**Type I error**	
***α***	***σ***_***Y***_	**QBlossoc**	**LSS**
5	10	0.03	0.03
5	20	0.06	0.07
5	30	0.04	0.10
5	40	0.03	0.03
7.5	10	0.06	0.04
7.5	20	0.06	0.06
7.5	30	0.05	0.05
7.5	40	0.07	0.06
10	10	0.08	0.03
10	20	0.04	0.07
10	30	0.03	0.03
10	40	0.02	0.03

For the values of *α* (the strength of selection) and *σ*_*Y*_ (the standard deviation of the quantitative trait per unit time) considered here, the type I error rates are all near 0.05 for these simulation results.

**Table 2:** Power and localization distance (bp) for simulated data sets

**Parameters**		**QBlossoc**		**LSS**	
***α***	***σ***_***Y***_	**Power**	**LocDist**	**Power**	**LocDist**
5	10	0.82	50524	0.78	56901
5	20	0.67	79306	0.64	94698
5	30	0.63	105197	0.61	130809
5	40	0.60	112477	0.60	134383
7.5	10	0.89	43247	0.90	25257
7.5	20	0.71	53905	0.74	44471
7.5	30	0.73	59379	0.64	96377
7.5	40	0.61	105529	0.58	123444
10	10	1.00	1593	0.98	5997
10	20	0.85	29476	0.84	30204
10	30	0.67	62315	0.73	68200
10	40	0.72	102386	0.65	82209

For the values of *α* (the strength of selection) and *σ*_*Y*_ (the standard deviation of the quantitative trait per unit time) considered here, the power and localization distance (in DNA base pairs) are comparable for QBlossoc and LSS.

Permutation testing results showed that both QBlossoc and LSS control the type I error around 0.05 (see Table 1). In terms of power of detection LSS is competitive with QBlossoc in this general case. The average localization distance (LocDist) is the shortest distance between the most significant (most highly-scored) SNP and the associated SNP in DNA base pairs. Smaller distances indicate a better statistic, and the two methods show approximately the same performance in terms of localization distance.

**Figure 3:**
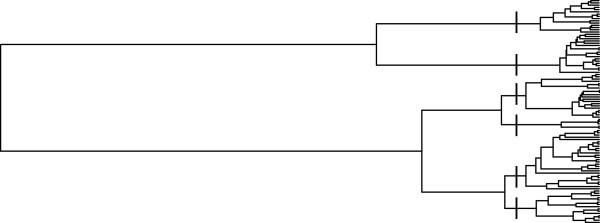
**Example of population structure.** This phylogenetic tree shows a form of population structure which impacts the correlation structure among the trait values. Six particular subpopulations are shown through the six groups of individuals present in the tree. The splits showing the six groups are denoted by hash marks. Notice that the underlying population structure is not necessarily shown directly in the evolutionary history of a SNP, but has a strong influence on the phylogenetic tree.

The special case of population stratification was also investigated. By using ms, population structures involving six subpopulations were specified. Here, the trees are constrained so that after a population splits into two subpopulations, no gene flow exists between the subsequent clades, and the subpopulations evolve independently of one another. This constraint is achieved through specification of times when the splits in populations occur, which are given below. Specifying the number of subpopulations and divergence times allows the investigation of the effect of the complexity of the population structure on the results. An example of a true phylogenetic tree for a particular replication with six specified subpopulations is shown in Figure 3.

**Figure 4:**
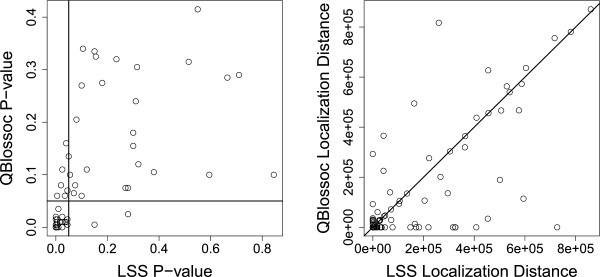
**Example of detection p-values and localization distances.** The left plot of the detection p-values and localization distance (in DNA bp) for one data set shows that QBlossoc and LSS are picking up different associations during simulation. In the left plot, values in the upper left and lower right corners of the plot show associations detected only by LSS and QBlossoc, respectively. Seven observations fall in the upper left corner, while two observations fall in the lower right corner of the plot. The sixty-five observations in the lower left corner were detected by both methods, while the remaining twenty-six observations were detected by neither method. The right plot shows the associations better localized by LSS and QBlossoc in the upper left and lower right regions, respectively. Twenty-three observations were better localized by LSS and forty-three observations were better localized by QBlossoc.

Results for selected studies are shown in Tables 3 and 4. In each case, six subpopulations were specified. The divergence times, or cumulative times at which population splits occurred, are (looking from present to past) 0.1, 0.1, 0.1, 0.2, and 1.0, and are omitted from Tables 3 and 4 for simplicity. These times are in evolutionary time units of 4*N*_0_*μ*=1.6×10^−5^ generations. The results in Table 3 show that the type I error is controlled by both QBlossoc and LSS in this special case. Also, the power of detection and localization distance (in DNA bp) are very similar for the two techniques (see Table 4). However, upon further investigation, we see that even though the two techniques have comparable powers of detection and average localization distances, QBlossoc and LSS are detecting different data sets. The left plot of Figure 4 shows the p-values for each simulated data set from the study with *α*=5 and *σ*_*Y*_=30 shown in Table 4. The horizontal and vertical lines represent the cutoff values for significance. The 65 observations in the lower left corner were detected by both QBlossoc and LSS, while the 26 observations in the upper right corner were detected by neither method. However, the seven observations in the upper left corner were detected by the proposed LSS but not QBlossoc, while the two observations in the lower right corner were detected by QBlossoc but not LSS. This is an indication that the proposed technique sometimes identifies different types of associations than QBlossoc.

**Table 3:** Type I error for simulated data sets showing population structure

**Parameters**		**Type I error**	
***α***	***σ***_***Y***_	**QBlossoc**	**LSS**
5	10	0.04	0.08
5	20	0.08	0.06
5	30	0.06	0.05
5	40	0.03	0.07
7.5	10	0.07	0.06
7.5	20	0.09	0.09
7.5	30	0.06	0.06
7.5	40	0.11	0.05
10	10	0.04	0.03
10	20	0.10	0.06
10	30	0.06	0.06
10	40	0.06	0.10

For the values of *α* (the strength of selection) and *σ*_*Y*_ (the standard deviation of the quantitative trait per unit time) considered here, the type I error rates are all near 0.05 for these simulation results in the case of six subpopulations. (Population divergence times for the six subpopulations are omitted for simplicity. See text for details).

**Table 4:** Power and localization distance for simulated data sets showing population structure

**Parameters**		**QBlossoc**		**LSS**	
***α***	***σ***_***Y***_	**Power**	**LocDist**	**Power**	**LocDist**
5	10	0.99	15048	0.98	13460
5	20	0.86	54969	0.86	65832
5	30	0.67	125523	0.72	152045
5	40	0.65	142156	0.63	179099
7.5	10	0.99	7877	0.98	11551
7.5	20	0.98	17076	0.97	24026
7.5	30	0.93	23691	0.89	39664
7.5	40	0.74	85356	0.71	124352
10	10	1.00	7375	1.00	12579
10	20	0.98	7428	0.99	15208
10	30	0.96	25168	0.95	21193
10	40	0.84	34017	0.87	44057

For the values of *α* (the strength of selection) and *σ*_*Y*_ (the standard deviation of the quantitative trait per unit time) considered here, the power and localization distance (in DNA base pairs) are comparable for QBlossoc and LSS in the case of six subpopulations. (Population divergence times for the six subpopulations are omitted for simplicity. See text for details).

Further, the right plot of Figure 4 shows the localization distances for each data set. Observations below the diagonal line indicate data sets in which QBlossoc was able to better localize the associated SNP, while observations above the diagonal line indicate data sets in which LSS was able to better localize the associated SNP. Twenty-three observations were better localized by LSS, while forty-three observations were better localized by QBlossoc. These simulation study results indicate that the proposed method is comparable with QBlossoc in the general case, and detecting different types of relationships between SNPs and quantitative traits in the case of population structure. Additionally, both QBlossoc and LSS appear to control the type I error in these simulation studies.

### Real data analysis

Having seen that the proposed method performs well for simulated data, we apply the method to a GWAS data set. The data set from [15] includes both SNP data and phenotypic data for 288 outbred mice. Phenotypic data for each mouse include observations about eight quantitative cardiovascular traits. Here, the trait we will focus on is the high-density lipoprotein cholesterol level (HDL). We will set *k*_*max*_=15 in LSS and use 200 permutations for the data analysis. The SNP sites on two chromosomes with previously-detected strong signals and one chromosome without a previously-detected strong signal are analyzed. In order to phase the data from genotypes into haplotypes, Beagle [20] was used, as in the original data analysis [15].

**Figure 5:**
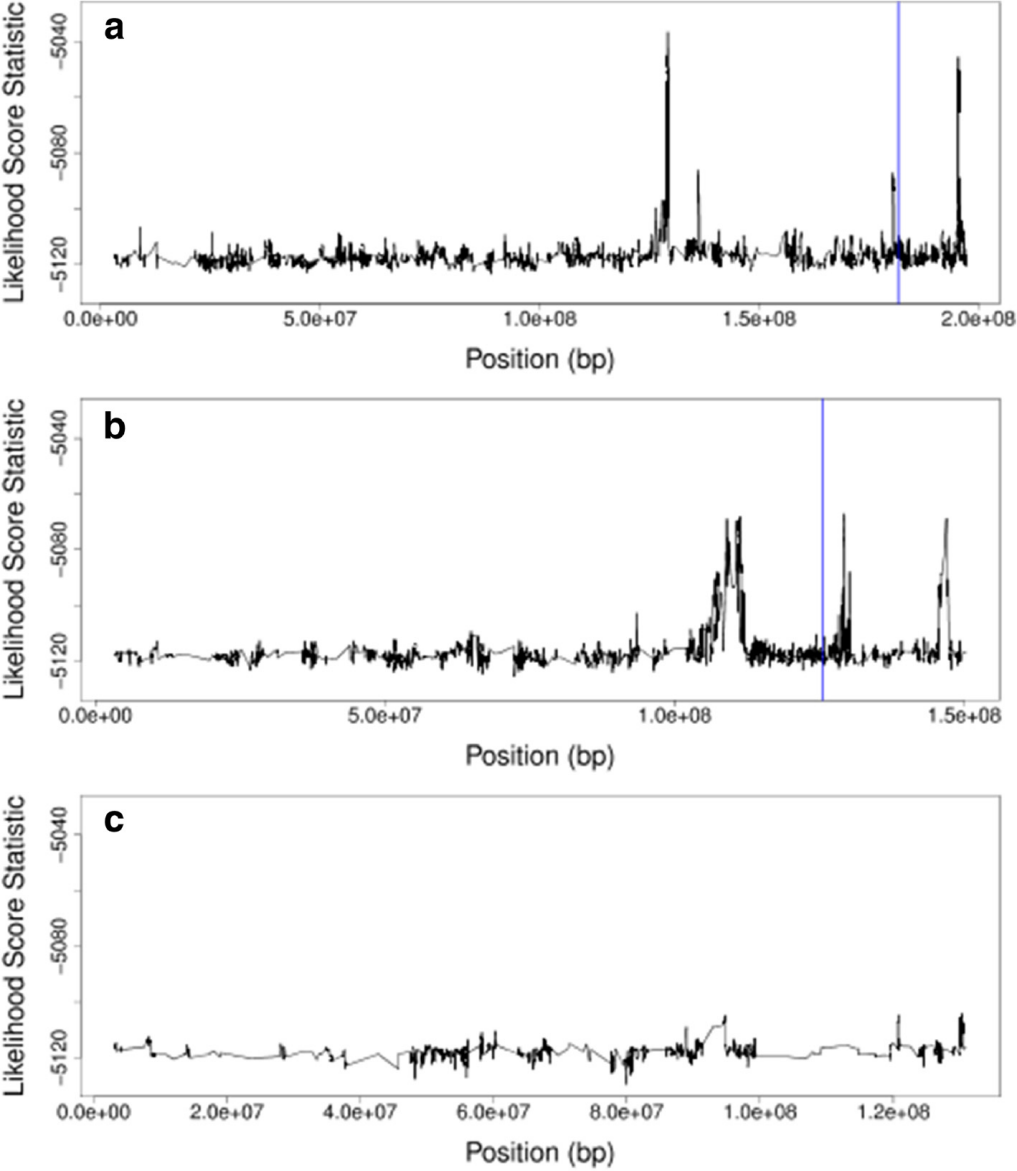
**Likelihood score statistic plot.** For the analysis of the HDL level in mice, this plot shows LSS plotted against the location of the SNP in DNA base pairs along Chromosomes 1, 5, and 8 in plots (**a**), (**b**), and (**c**), respectively. The vertical lines represent locations detected as highly significant by Zhang et al. [15].

Chromosome 1 included data for 4,165 SNPs, and was analyzed using the proposed method. The method detected the chromosome with a p-value less than 0.005. The score results, presented in Figure 5(a), show that of the two sites detected as highly significant by [15], LSS shows a peak very near to one of these sites. In addition, three other sites not previously detected show very large peaks in LSS. These results support the simulation study results, in which LSS tends to detect different signals than previous methods.

Zhang et al. [15] also found a strong genetic signal on Chromosome 5. Chromosome 5 included data for 3,185 SNPs, and was analyzed using the proposed method. The method detected the chromosome with a p-value less than 0.005. The results presented in Figure 5(b), show a peak in LSS near the SNP site previously detected as highly significant [15]. In addition, two other regions on the chromosome not previously detected show very large peaks in LSS.

Chromosome 8 was also analyzed, and results are presented in Figure 5(c). Chromosome 8 included data for 1,159 SNP sites. Zhang et al. [15] did not detect any highly significant SNP sites on Chromosome 8. The likelihood analysis resulted in a detection p-value of 0.055 for this chromosome, which is not significant.

## Discussion and conclusion

Here, a method is presented to search for SNPs associated with quantitative traits in GWAS data. The proposed method is a modification of QBlossoc which relaxes the assumptions of independence and common variance between observations. The proposed method looks at this problem using a framework which accounts for the evolutionary relationships among SNPs. However, as opposed to previous techniques using these evolutionary relationships, the method here remains computationally feasible by using only the broad-scale relationships present in the evolutionary history among SNPs. These evolutionary relationships impact results especially in the presence of strong population structure.

Using an innovative, biologically-sensible technique, simulated data sets were obtained in both the general case and in the presence of population structure. Simulation results showed that LSS is competitive with QBlossoc in terms of localization and power of detection, and that different chromosomes may be detected by LSS and by QBlossoc. In the presence of population stratification, the proposed score shows particularly strong performance. For the real data example studying 288 outbred mice, analysis using the proposed tree estimation and likelihood score showed that LSS detects two SNPs previously linked to HDL in mice. In addition, LSS also detected several SNPs not previously mentioned in the literature.

One of the advantages of this proposed method is its use of ancestral information to approach this problem. This framework is more realistic than other previous approximations. Also, the use of the broad-scale evolutionary relationships among SNPs makes the technique computationally feasible. Computation times for the branch length estimation and LSS analysis, including permutation testing, ranged from approximately 3.5 to 5.5 seconds per SNP on a standard desktop linux machine for the simulated data sets with 100 observations, which typically included between 65 and 105 SNPs. For the real data analysis, with 576 observations, these computation times ranged from approximately 8 to 35 minutes per SNP, depending on the number of SNPs along the chromosome (ranging from 1,159 for Chromosome 8 to 4,165 for Chromosome 1). It should be noted that individual SNP computations are easily parallelized in this setting.

Although the proposed technique begins to address the limitations of current statistical methodology in the problem of quantitative trait mapping, the technique has several avenues that could be pursued in order to extend the method to more general cases. In the data simulation technique, only codominant trait models have been implemented, but dominant and recessive trait models are straightforward to implement and test. Also, many traits are impacted by both a genetic component and an environmental covariate. By extending the quantitative trait simulation technique, many realistic traits could be simulated with both genetic and environmental covariates.

Similarly, LSS is flexible and could be generalized to include environmental covariates as well. Additionally, the current likelihood score requires that genotypic data be phased into haplotypes prior to analysis. Phasing is a nontrivial process which is subject to error. By extending the tree estimation method and likelihood score to be computed on genotypic data, these methods will be more easily applied to real data sets. Advantages of the model include its ability to find different signals than previous statistical methods and its flexibility to be extended to analyze different types of data. Although these extensions are under investigation, the proposed data analysis technique appears to be an impactful modification of the ideas presented in QBlossoc, especially in the presence of population structure.

## Competing interests

Both authors have no competing interests.

## Authors’ contributions

Both authors developed the methodology. KLT coded the algorithm, carried out all analyses, and drafted the manuscript. LSK revised the manuscript. Both authors read and approved the final manuscript.

## References

[B1] McClurg P Pletcher TM Wiltshire T Su AI Comparative analysis of haplotype association mapping algorithms BMC Bioinformatics 2006 7 61 10.1186/1471-2105-7-61 16466585 PMC1409800

[B2] Zhang Z Zhang X Wang W HTreeQA: Using semi-perfect phylogeny trees in quantitative trait loci study on genotype data G3: Genes, Genomes, Genetics 2012 2 175 189 10.1534/g3.111.001768 PMC3284325 22384396

[B3] Roses AD Post-GWAS: Phylogenetic analysis in the hunt for complex disease-associated loci J Pharmacogenomics Pharmacoproteomics 2012 3 3

[B4] Zöllner S Pritchard JK Coalescent-based association mapping and fine mapping of complex trait loci Genetics 2005 169 1071 1092 10.1534/genetics.104.031799 15489534 PMC1449137

[B5] Minichiello MJ Durbin R Mapping trait loci by use of inferred ancestral recombination graphs Am J Human Genet 2006 79 910 922 10.1086/508901 17033967 PMC1698562

[B6] Mailund T Besenbacher S Schierup MH Whole genome association mapping by incompatibilities and local perfect phylogenies BMC Bioinformatics 2006 7 454 10.1186/1471-2105-7-454 17042942 PMC1624851

[B7] Pan F McMillan L de Villena FPM Threadgill D Wang W TreeQA: Quantitative genome wide association mapping using local perfect phylogeny trees Pacific Symposium on Biocomputing 2009 415 426 http://www.ncbi.nlm.nih.gov/pmc/articles/PMC2739990/pdf/nihms132006.pdf 19209719 PMC2739990

[B8] Besenbacher S Mailund T Schierup MH Local phylogeny mapping of quantitative traits: higher accuracy and better ranking than single-marker association in genomewide scans Genetics 2009 181 747 753 19064712 10.1534/genetics.108.092643 PMC2644962

[B9] Wakeley J Coalescent Theory: An Introduction 2009 Colorado: Roberts & Company Publishers

[B10] Kingman JFC The coalescent Stochastic Processes Appl 1982 13 235 248 10.1016/0304-4149(82)90011-4

[B11] Wang L Zhang K Zhang L Perfect phylogenetic networks with recombination J Comput Biol 2001 8 69 78 10.1089/106652701300099119 11339907

[B12] Wu Y New methods for inference of local tree topologies with recombinant SNP sequences in populations IEEE/ACM TCBB 2011 8 182 193 21071806 10.1109/TCBB.2009.27

[B13] Rogers JS Swofford DL A fast method for approximating maximum likelihoods of phylogenetic trees from nucleotide sequences Syst Biol 1998 47 77 89 10.1080/106351598261049 12064242

[B14] Felsenstein J Brownian motion and gene frequencies Inferring Phylogenies 2004 Massachusetts: Sinauer Associates, Inc. 391 414

[B15] Zhang W Korstanje R Thaisz J Staedtler F Harttman N Xu L Feng M Yanas L Yang H Valdar W Churchill GA DiPetrillo K Genome-wide association mapping of quantitative traits in outbred mice G3 (Bethesda) 2012 2 2 167 174 2012 22384395 10.1534/g3.111.001792 PMC3284324

[B16] Jukes TH Cantor CR Evolution of Protein Molecules 1969 New York: Academic Press

[B17] Lewis PO A likelihood approach to estimating phylogeny from discrete morphological character data Syst Biol 2001 50 6 913 925 10.1080/106351501753462876 12116640

[B18] Hudson RR Generating samples under a Wright-Fisher neutral model of genetic variation Bioinformatics 2002 18 337 338 10.1093/bioinformatics/18.2.337 11847089

[B19] Hansen TF Stabilizing selection and the comparative analysis of adaptation Evolution 1997 51 5 1341 1351 10.2307/2411186 28568616

[B20] Browning SR Browning BL Rapid and accurate haplotype phasing and missing data inference for whole genome association studies using localized haplotype clustering Am J Human Genet 2007 81 1084 1097 10.1086/521987 17924348 PMC2265661

